# Study on copy number variant detection results of 472 fetuses with non-structural ultrasound abnormalities in non-advanced age pregnant women and the correlation with adverse obstetric history

**DOI:** 10.3389/fgene.2026.1895948

**Published:** 2026-10-30

**Authors:** Lifang Lin, Heming Wu, Zhiyuan Zheng, Ping Chen, Jianwen Lian, Shuxian Huang, Liubing Lan

**Affiliations:** 1 Department of Prenatal Diagnostic Center, Meizhou People’s Hospital, Meizhou Academy of Medical Sciences, Meizhou, China; 2 Meizhou Municipal Engineering and Technology Research Center for Molecular Diagnostics of Major Genetic Disorders, Meizhou People’s Hospital, Meizhou Academy of Medical Sciences, Meizhou, China; 3 Department of Ultrasound, Meizhou People’s Hospital, Meizhou Academy of Medical Sciences, Meizhou, China; 4 Department of Obstetrics, Meizhou People’s Hospital, Meizhou Academy of Medical Sciences, Meizhou, China

**Keywords:** adverse obstetric history, chromosomal microarray analysis, copy number variant, non-advanced maternal age, non-structural ultrasound abnormalities, ultrasound soft marker

## Abstract

**Objective:**

To investigate copy number variant (CNV) results in 472 fetuses with non-structural ultrasound abnormalities among pregnant women under 35 years, and analyze the association between adverse obstetric history and pathogenic/likely pathogenic (P/LP) CNVs and variants of uncertain significance (VUS).

**Methods:**

A total of 472 pregnant women aged <35 years with fetal non-structural ultrasound abnormalities were retrospectively enrolled. All participants were divided into groups with or without adverse obstetric history. We summarized the distribution of non-structural ultrasound abnormalities, calculated the overall and stratified detection rates of P/LP CNVs and VUS, and compared differences between groups.

**Results:**

The mean maternal age was 28.73 ± 3.59 years; 22.0% had a positive history. Abnormal ultrasound soft markers accounted for 82.6% of cases, followed by fetal growth restriction (12.3%) and abnormal amniotic fluid volume (4.0%). The overall detection rates were 5.7% for P/LP CNVs and 9.1% for VUS. The highest detection rate of P/LP CNVs was observed in fetuses with abnormal amniotic fluid volume (10.5%), while the highest detection rate of VUS was seen in abnormal ultrasound soft markers (9.2%). The combined detection rate of P/LP and VUS differed among different types of isolated abnormal ultrasound soft markers, with tricuspid regurgitation, persistent right umbilical vein and persistent left superior vena cava showing higher detection rates. No significant differences in overall P/LP CNV and VUS rates were found between women with and without adverse pregnancy history (*p* > 0.05).

**Conclusion:**

Non-advanced age pregnant women with fetal non-structural ultrasound abnormalities carry a non-negligible risk of P/LP CNVs and VUS. Different ultrasound soft markers present distinct CNV detection rates. Adverse obstetric history does not alter the P/LP CNVs and VUS detection rate.

## Introduction

With the rapid development of prenatal ultrasound technology and genetic detection techniques, prenatal screening and diagnosis have become core components of modern obstetric care for preventing birth defects (Wu et al., 2025). Ultrasound serves as a conventional tool in prenatal diagnosis for initial evaluation of fetal development and identification of suspected anomalies (Chapman et al., 2026; Daly et al., 2026). As a standard approach for fetal malformation screening, prenatal ultrasonography has gained extensive clinical application. It is capable of revealing a variety of fetal issues, such as structural defects, mild anomalies (ultrasound soft markers), fetal growth restriction (FGR) and abnormal amniotic fluid volume (Chen et al., 2024). All ultrasonic abnormalities other than structural anomalies are collectively defined as non-structural ultrasound abnormalities (Liubing et al., 2024; Hui et al., 2025). Fetal structural abnormalities can be easily identified by routine prenatal ultrasound, while non-structural ultrasound abnormalities, refer to subtle ultrasonic manifestations that do not constitute definite organic malformations but are potentially associated with fetal chromosomal abnormalities and genetic variations (Liubing et al., 2024; Peng et al., 2024). These markers are frequently detected in routine prenatal ultrasound examinations and bring considerable clinical anxiety and counseling challenges to pregnant women and clinicians.

Copy number variants (CNVs) are defined as genomic DNA segment changes including deletions, duplications, insertions and complex multi-copy rearrangements, with the fragment length exceeding 1 kilobase (Kb) (Lan et al., 2025). Scattered throughout the human genome, CNVs constitute a primary type of genomic structural variation (Wu et al., 2024). They interfere with embryonic growth and human physiological activities via diverse molecular pathways, such as changing gene dosage, destroying gene integrity and modulating the expression of neighboring genes (Abu-Elmagd et al., 2022; Malwade et al., 2025). Pathogenic or likely pathogenic (P/LP) CNVs are important genetic contributors to fetal developmental defects, poor pregnancy outcomes and neonatal congenital disorders (Ren et al., 2025; Zheng et al., 2026). Chromosomal microarray analysis (CMA) enables high-resolution detection of CNVs in tiny chromosomal regions through whole-genome screening (Liubing et al., 2024; Karataş et al., 2025). Compared with traditional karyotype analysis, CMA possesses higher resolution and can effectively detect micro-deletions and micro-duplications that cannot be identified by conventional cytogenetic methods (Liubing et al., 2024). It has been recommended as a first-tier genetic detection technique for prenatal fetuses with ultrasound abnormalities.

At present, clinical studies have confirmed that fetal non-structural ultrasound abnormalities are closely correlated with the risk of fetal CNVs (Chen et al., 2024; Liubing et al., 2024; Cai et al., 2020). However, different types of non-structural ultrasound abnormalities vary greatly in the positive rate of CNV detection, and the clinical significance of most non-structural ultrasound abnormalities remains controversial (Cai et al., 2020; Chen et al., 2021). Moreover, most existing studies focus on the relationship between ultrasound markers and fetal genetic abnormalities, while fewer investigations specifically concentrate on non-advanced age pregnant women who are traditionally considered to have a low risk of chromosomal anomalies. Clinicians often lack sufficient genetic evidence for individualized prenatal counseling for non-advanced age pregnant women with fetal non-structural ultrasound abnormalities.

A history of adverse pregnancy, including recurrent spontaneous abortion, stillbirth, fetal malformation, and adverse neonatal outcomes, is an important high-risk factor for recurrent fetal genetic abnormalities and adverse pregnancy prognosis (Liang J. et al., 2026). Whether maternal adverse obstetric history can affect the detection rate of fetal P/LP CNVs and VUS in non-advanced age pregnant women with fetal non-structural ultrasound abnormalities remains unclear, and relevant evidence-based data are still insufficient. Therefore, this study retrospectively enrolled 472 singleton fetuses with non-structural ultrasound abnormalities from non-advanced age pregnant women. We aimed to analyze the overall distribution characteristics of different non-structural ultrasound abnormalities, compare the CNV detection yield among various ultrasonic soft markers, and further explore the correlation between maternal adverse obstetric history and fetal CNV detection results. The findings are expected to provide objective clinical evidence for prenatal genetic counseling, rational selection of prenatal genetic detection, and clinical management for non-advanced age pregnant women with fetal non-structural ultrasound abnormalities.

## Materials and methods

### Study cohort

This retrospective observational study enrolled a total of 472 non-advanced age pregnant women with fetuses presenting non-structural ultrasound abnormalities who underwent prenatal genetic testing at Meizhou People’s Hospital between October 2022 and December 2025. All enrolled pregnant women were aged <35 years and categorized as non-advanced age pregnant women.

Inclusion criteria were as follows (Wu et al., 2025): maternal age <35 years old (Chapman et al., 2026); prenatal ultrasound indicated fetal non-structural abnormalities/ultrasound soft markers without definite structural malformations (Daly et al., 2026); complete clinical ultrasound data and prenatal genetic testing results; and (Chen et al., 2024) complete baseline information including maternal age, gestational age at sampling, and adverse obstetric history. Exclusion criteria were as follows (Wu et al., 2025): advanced maternal age (≥35 years old) (Chapman et al., 2026); fetal definite structural malformation detected by ultrasound (Daly et al., 2026); multiple pregnancy (Chen et al., 2024); fetuses with chromosome aneuploidies or structural chromosome aberrations revealed by karyotype analysis (Liubing et al., 2024); incomplete clinical, ultrasound or genetic detection data; and (Hui et al., 2025) pregnant women with known chromosomal abnormalities, familial hereditary diseases, or severe systemic organic diseases.

The study was performed under the guidance of the Declaration of Helsinki and approved by the Ethics Committee of Medicine, Meizhou People’s Hospital (Clearance No.: 2024-C-92). All participants were fully informed of the purpose, detection process and clinical significance of prenatal genetic testing, and signed written informed consent. Unified case record forms were used to collect baseline data, including maternal age, gestational age at sampling, adverse pregnancy history, types of fetal specimens, and classification of fetal non-structural ultrasound abnormalities. All clinical and genetic data were anonymized prior to analysis: all identifiable personal identifiers including name, ID number, contact information were removed, and each case was assigned an independent study-only case ID number. No identifiable personal information was retained in the study database.

All data were double input and checked to ensure accuracy and consistency. According to the presence or absence of adverse pregnancy history, all pregnant women were divided into two groups: without and with adverse obstetric history groups. Adverse obstetric history was defined as previous occurrences of spontaneous abortion, induced abortion due to fetal abnormality, stillbirth, neonatal death, or delivery of offspring with congenital malformations and chromosomal diseases. Fetal non-structural ultrasound abnormalities were classified into four categories: abnormal ultrasound soft markers, fetal growth restriction, abnormal amniotic fluid volume, and cases with more than two combined categories.

### Classification of fetal non-structural ultrasound abnormalities

#### Ultrasound soft marker abnormalities

Common abnormal soft markers detected by fetal ultrasound encompass a variety of subtle phenotypic anomalies, including enlarged nuchal translucency (NT), cerebral ventriculomegaly, nasal bone underdevelopment, choroid plexus cysts, shortened long bones, renal pelvic dilation, hyperechoic bowel, subependymal cysts, aberrant subclavian artery, single umbilical artery, tricuspid valve regurgitation, persistent left superior vena cava, persistent right umbilical vein, thickened nuchal fold (NF), and intracardiac hyperechoic foci. All non-structural fetal ultrasound abnormalities and soft markers were initially identified by the attending sonographer, and each finding was further confirmed by a second experienced prenatal ultrasound physician prior to invasive prenatal sampling as part of standard clinical practice. The standardized diagnostic criteria for the above fetal ultrasound soft markers are specified as follows (Hu et al., 2021; Pan et al., 2023):

1. Increased NT thickness: Nuchal translucency measurement of ≥3.0 mm in fetuses at 11 to 13^+6^ weeks of gestation.
2. Ventriculomegaly: A transverse diameter of the lateral ventricular atrium exceeding 10 mm at any gestational stage.
3. Nasal bone hypoplasia: Ultrasonic manifestation of unobservable ossified nasal bone or abnormally shortened nasal bone length.
4. Choroid plexus cyst: Avascular anechoic cystic lesions detected in the hyperechoic choroid plexus of fetal lateral ventricles during 16–24 weeks of pregnancy.
5. Short long bones: Fetal femoral or humeral length falling below the 10th percentile of the normal reference range for the corresponding gestational age.
6. Renal pelvic dilatation: The anteroposterior diameter of the fetal renal pelvis ranging from 0.6 cm to 1.0 cm.
7. Echogenic bowel: Intestinal tissue echoes that are equal to or higher in signal intensity than the adjacent fetal skeletal echoes on ultrasound imaging.
8. Subependymal cysts: Well-defined avascular anechoic cystic lesions beneath the ventricular ependyma, with no subsequent progressive ventricular dilatation.
9. Aberrant right subclavian artery: A vascular malformation where the right subclavian artery originates from the distal segment of the proximal descending aorta, courses behind the trachea, and develops after the branching of the left common carotid artery and left subclavian artery from the aortic arch.
10. Single umbilical artery: Ultrasonic visualization of only one umbilical artery adjacent to the fetal bladder.
11. Tricuspid regurgitation: Systolic retrograde blood flow from the right ventricle to the right atrium across the tricuspid valve, as detected by Doppler echocardiography in the apical four-chamber view.
12. Persistent left superior vena cava: A congenital venous anomaly caused by the failure of embryonic regression of the left common cardinal vein.
13. Persistent right umbilical vein: A fetal vascular variation characterized by patent right umbilical vein and obliterated left umbilical vein.
14. Increased nuchal fold (NF): Nuchal fold thickness of 6 mm or more in fetuses beyond 15 weeks of gestation.
15. Echogenic intracardiac focus: Isolated punctate hyperechoic lesions within the fetal ventricular cavity, with echo intensity consistent with that of fetal bony tissue.

#### Amniotic fluid abnormalities

Abnormalities in amniotic fluid volume primarily include polyhydramnios and oligohydramnios. Polyhydramnios is diagnosed when the deepest vertical pocket (DVP) measures 8 cm or greater and/or the amniotic fluid index (AFI) is 24 cm or higher. In contrast, oligohydramnios is confirmed by a DVP of 2 cm or less and/or an AFI lower than 8 cm (Shi et al., 2023; Simonyi et al., 2021).

#### Fetal growth restriction

Fetal growth restriction refers to a condition where the ultrasonographically estimated fetal weight falls below the 10th percentile of the standard range for the corresponding gestational age, which may be accompanied by anomalous Doppler parameters of the umbilical artery or middle cerebral artery, or present as an isolated anomaly (Rottenstreich et al., 2026).

Fetal non-structural ultrasound anomalies are a comprehensive category consisting of atypical ultrasonic soft markers, fetal growth restriction, and amniotic fluid volume disorders (Luo et al., 2021; Huang et al., 2022). All enrolled fetuses were categorized based on prenatal ultrasound findings into four groups: isolated abnormal ultrasound soft markers, isolated fetal growth restriction, isolated abnormal amniotic fluid volume, and cases with two or more combined non-structural ultrasound abnormalities.

### Sample collection and processing

Prenatal sampling was performed according to gestational age: chorionic villus sampling was conducted at 11–13^+6^ weeks of gestation, amniocentesis at 14–28 weeks, and umbilical cord blood puncture after 28 weeks of gestation. Nevertheless, amniocentesis could still be performed between 12–13^+6^ weeks and beyond 28 weeks under certain clinical conditions when chorionic villus sampling and umbilical cord blood puncture were not feasible. Aseptic operation was strictly followed during the sampling process to avoid contamination. Strict management was applied to the storage, transportation and processing of all samples in a standard lab setting, which protected DNA from degradation and exogenous contamination. Fetal genomic DNA was extracted using the Qiagen DNA extraction kit (Qiagen, Valencia, CA, USA). DNA concentration and purity were quantified using Qubit fluorometer (Thermo Fisher Scientific, USA), and samples with DNA concentration ≥50 ng/μL and A260/A280 ratio between 1.8–2.0 were qualified for subsequent testing. Quantitative fluorescence polymerase chain reaction (QF-PCR) using commercialized diagnostic test kit (Darui Biotechnology Co., Ltd., Guangzhou, China), performed on an GenReader 7010 Genetic analyzer (Microread Genetics Co., Ltd., Beijing, China) to exclude maternal cell contamination for all specimens according to manufacturer-standard protocols.

### CNV detection and classification

CMA was applied to detect fetal CNVs. Genomic DNA was extracted from villus tissue, amniotic fluid cells or umbilical cord blood samples. Restriction enzyme NspI was used to cleave genomic DNA into small fragments. Upon amplification with biotin labeling, the DNA specimens were combined with hybridization solution completely and denatured. These samples were then hybridized onto CytoScan 750K arrays, followed by washing, staining and scanning according to the manufacturer’s standard workflow. Raw CEL files generated by chip scanning were imported into Chromosome Analysis Suite (ChAS, version 4.3) for primary data normalization, QC filtering and CNV calling. The reference genome build was GRCh37 (hg19), and NetAffx annotation database version 35.1 was adopted. QC filtering thresholds were set as follows: MAPD ≤0.25, SNP-QC ≥15. For CNV reporting, the minimum probe coverage threshold was ≥50 contiguous array probes; the minimum reportable size was ≥400 Kb for duplications and ≥400 Kb for deletions. The approximate lower detection limit for mosaicism under satisfactory sample QC was about 20%. Regions of absence of heterozygosity (AOH)/regions of homozygosity (ROH) were evaluated; candidate uniparental disomy (UPD) events were flagged from large autosomal ROH segments. Sex-chromosome copy-number and SNP-allele patterns were fully assessed for all study samples.

Variant classification was performed following the 2020 American College of Medical Genetics and Genomics (ACMG) technical standards for constitutional copy-number variants. Multiple lines of evidence were systematically evaluated, including variant size, gene content, presence of dosage-sensitive genes, population frequency from Database of Genomic Variants (DGV), clinical phenotype data from Database of Genomic Variation and Phenotype in Humans using Ensembl Resources (DECIPHER) and Clinical Genome Resource (ClinGen), Online Mendelian Inheritance in Man (OMIM), and published literature (PubMed). Each CNV was assigned to one of five categories: pathogenic (P), likely pathogenic (LP), variants of uncertain significance (VUS), likely benign (LB), and benign (B), based on the weighted combination of available evidence (Riggs et al., 2020; Brandt et al., 2020). It should be noted that the P/LP CNV reported in the present study only includes submicroscopic deletions and duplications. Chromosome numerical aneuploidies were excluded according to our pre-defined exclusion criteria and were not incorporated into the P/LP CNV statistics. P/LP CNV was set as the primary endpoint, VUS was treated as a secondary exploratory outcome. The combined rate of P/LP + VUS was provided only for supplementary description of overall detection yield and does not represent pathogenic genetic risk.

### Statistical analysis

Statistical analysis in this study was carried out via SPSS. Numbers and percentages were used to show enumeration data. Differences in the prevalence of P/LP CNVs and VUS were analyzed by χ^2^ test or Fisher’s exact test. Specifically, Fisher’s exact test was applied when >20% of cells in the contingency table had expected frequencies below five or when the total sample size of the subgroup was less than 40; otherwise, the χ^2^ test was adopted for categorical variable comparison. To control family-wise type I error resulting from multiple subgroup comparisons across different ultrasound soft-marker subtypes, Bonferroni correction was applied. The adjusted *p* value was calculated by dividing the original significance threshold (α = 0.05) by the number of independent statistical tests. All reported *p* values in Table 1 are Bonferroni-adjusted *p* values. Differences with *p* < 0.05 were regarded as statistically significant.

**TABLE 1:** Comparison the detection rates of P/LP CNVs, VUS in fetuses of pregnant women without and with adverse obstetric history among different types of single ultrasonic soft marker abnormalities.

Single ultrasonic soft marker abnormalities	Number of cases		P/LP CNVs				VUS			
	Without adverse obstetric history (n = 277)	With adverse obstetric history (n = 82)	Without adverse obstetric history	With adverse obstetric history	p values	95% confidence interval	Without adverse obstetric history	With adverse obstetric history	p values	95% confidence interval
Increased NT thickness (n = 128)	102	26	4 (3.9%)	2 (7.7%)	0.601	0.596–0.615	11 (10.8%)	2 (7.7%)	0.738	0.732–0.749
Ventriculomegaly (n = 69)	54	15	3 (5.6%)	1 (6.7%)	1.000	1.000–1.000	4 (7.4%)	0 (0)	0.570	0.567–0.586
Nasal bone hypoplasia (n = 53)	43	10	2 (4.7%)	0 (0)	1.000	1.000–1.000	3 (7.0%)	0 (0)	0.615	0.603–0.622
Choroid plexus cyst (n = 37)	20	17	0 (0)	1 (5.9%)	0.459	0.444–0.464	2 (10.0%)	1 (5.9%)	1.000	1.000–1.000
Short long bones (n = 23)	22	1	1 (4.5%)	0 (0)	1.000	1.000–1.000	4 (18.2%)	0 (0)	1.000	1.000–1.000
Pyelic separation (n = 16)	13	3	1 (7.7%)	0 (0)	1.000	1.000–1.000	3 (23.1%)	0 (0)	0.582	0.570–0.589
Echogenic bowel (n = 13)	9	4	0 (0)	1 (25.0%)	0.308	0.293–0.311	0 (0)	2 (50.0%)	0.077	0.071–0.081
Bilateral subependymal cysts (n = 5)	4	1	0 (0)	0 (0)	-	-	1 (25.0%)	0 (0)	1.000	1.000–1.000
Aberrant subclavian artery (n = 5)	3	2	0 (0)	0 (0)	-	-	0 (0)	0 (0)	-	-
Single umbilical artery (n = 3)	3	0	0 (0)	-	-	-	0 (0)	-	-	-
Tricuspid regurgitation (n = 3)	0	3	-	2 (66.7%)	-	-	-	0 (0)	-	-
Persistent left superior vena cava (n = 2)	2	0	0 (0)	-	-	-	1 (50.0%)	-	-	-
Persistent right umbilical vein (n = 2)	2	0	0 (0)	-	-	-	1 (50.0%)	-	-	-

CNV, copy number variant; P/LP CNV, Pathogenic/Likely pathogenic CNV; VUS, variants of uncertain significance.

This table only includes subjects with a single isolated ultrasonic soft marker abnormality. Cases with two or more concurrent soft marker abnormalities (n = 31) were excluded from these subgroup comparisons.

## Results

### Baseline characteristics of study population

A total of 472 pregnant women with fetal non-structural ultrasound abnormalities were enrolled in the present study. The mean maternal age was 28.7 ± 3.6 years. Of all participants, 368 (78.0%) had no adverse obstetric history, while 104 (22.0%) reported a previous adverse pregnancy history.

According to the gestational age at sampling, 60 cases (12.7%) were sampled at ≤13 weeks of gestation, 325 cases (68.9%) at 14–28 weeks, and 87 cases (18.4%) at over 28 weeks. In terms of fetal specimen types, chorionic villus samples accounted for 12.5% (59/472), amniotic fluid samples accounted for 87.3% (412/472), and umbilical cord blood samples accounted for only 0.2% (1/472) (Table 2).

**TABLE 2:** General characteristics of pregnant women and the fetuses.

Characteristics	Cases (n = 472)
Age of pregnant women (years)	28.7 ± 3.6
Adverse obstetric history	​
No, n (%)	368 (78.0%)
Yes, n (%)	104 (22.0%)
The gestational age at the time of sampling (weeks)	​
≤13, n (%)	60 (12.7%)
14–28, n (%)	325 (68.9%)
>28, n (%)	87 (18.4%)
Types of fetuses samples	​
Villus, n (%)	59 (12.5%)
Amniotic fluid, n (%)	412 (87.3%)
Umbilical cord blood	1 (0.2%)

### Composition classification of fetal non-structural ultrasound abnormalities

Among the 472 fetuses with non-structural ultrasound abnormalities, abnormal ultrasound soft markers were the most common subtype, accounting for 82.6% (390/472). Fetal growth restriction was observed in 12.3% (58/472) of cases, abnormal amniotic fluid volume in 4.0% (19/472), and more than two combined non-structural ultrasound abnormalities in 1.1% (5/472) (Figure 1).

**FIGURE 1:**
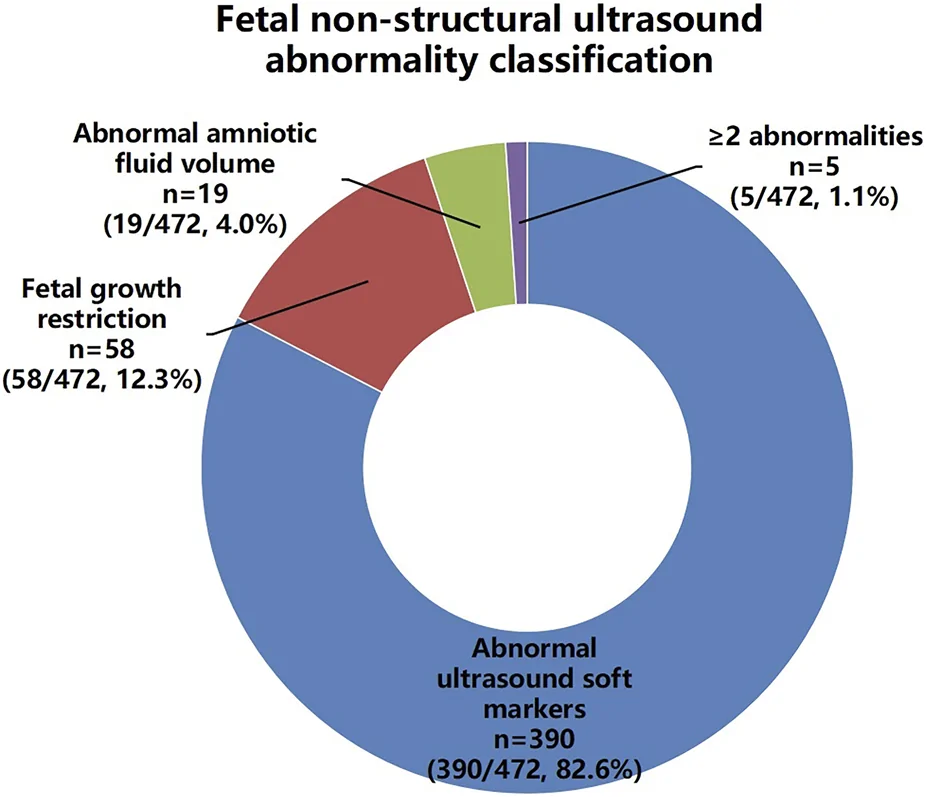
Fetal non-structural ultrasound abnormality classification.

### Overall detection of fetal CNVs

The overall detection results of CNVs were shown in Figure 2. Pathogenic/Likely pathogenic (P/LP) CNVs were detected in 27 cases, with a detection rate of 5.7%. VUS were identified in 43 cases, accounting for 9.1%. No P/LP CNVs or VUS CNVs were detected in 402 cases (85.2%) of the total cohort. Detailed information of 27 fetuses with P/LP CNVs and 43 fetuses with VUS detected by CMA analysis are presented in Supplementary Table S1 and Supplementary Table S2.

**FIGURE 2:**
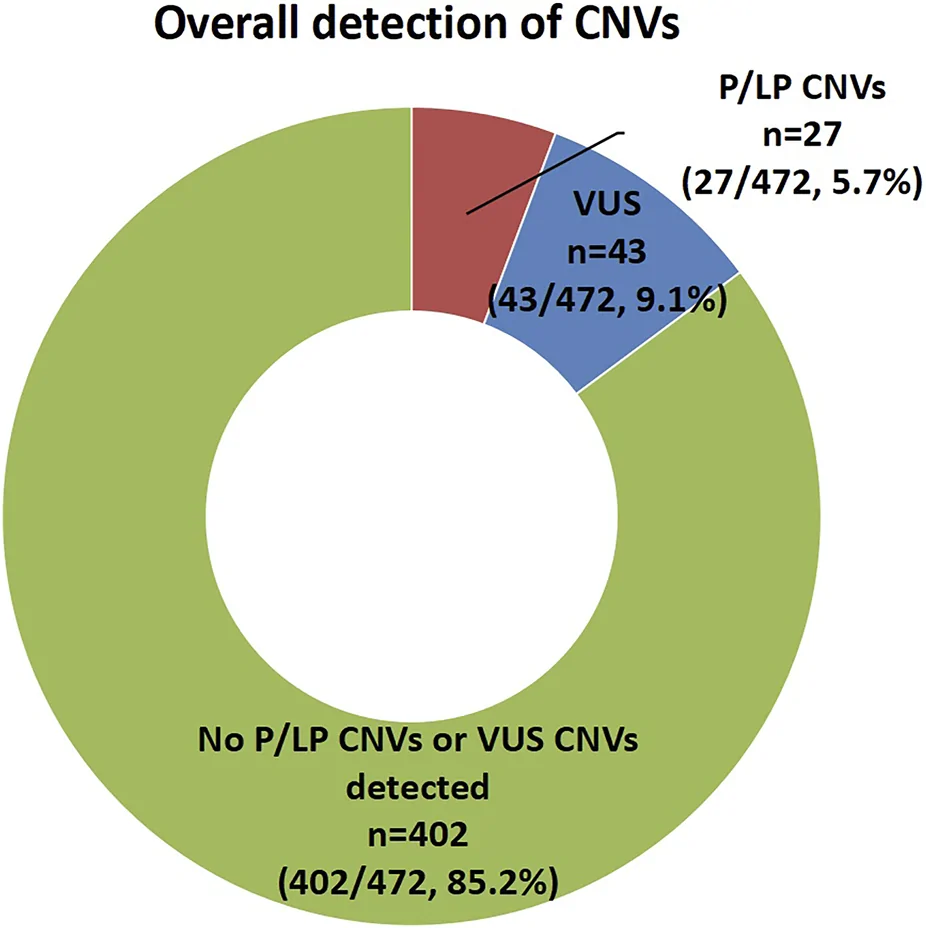
Overall detection of CNVs. Note: CNV, copy number variant; P/LP CNV, Pathogenic/Likely pathogenic CNV; VUS, variants of uncertain significance.

### CNV detection rates stratified by non-structural ultrasound abnormality subtypes

Stratified analysis based on fetal non-structural ultrasound abnormality classifications revealed distinct CNV detection rates across different subgroups (Figure 3). Overall, the detection rates of CNVs varied across different categories of fetal non-structural ultrasound abnormalities, with distinct patterns observed between P/LP CNVs and VUS, although the difference was not statistically significant (*p* = 0.819, 95% confidence interval (CI): 0.811–0.826). For P/LP CNVs, the highest detection rate was observed in fetuses with abnormal amniotic fluid volume (10.5%), followed by those with fetal growth restriction (8.6%), and the lowest rate was found in cases with abnormal ultrasound soft markers (4.9%). The difference was not statistically significant (*p* = 0.268, 95% CI: 0.260–0.277). For VUS, the highest detection rate was seen in fetuses with abnormal ultrasound soft markers (9.2%), followed by fetal growth restriction (8.6%), and the lowest rate was noted in cases with abnormal amniotic fluid volume (5.3%). The difference was not statistically significant (*p* = 0.895, 95% CI: 0.889–0.901). Notably, the detection rate of VUS was equal to that of P/LP CNVs in the fetal growth restriction group (both 8.6%).

**FIGURE 3:**
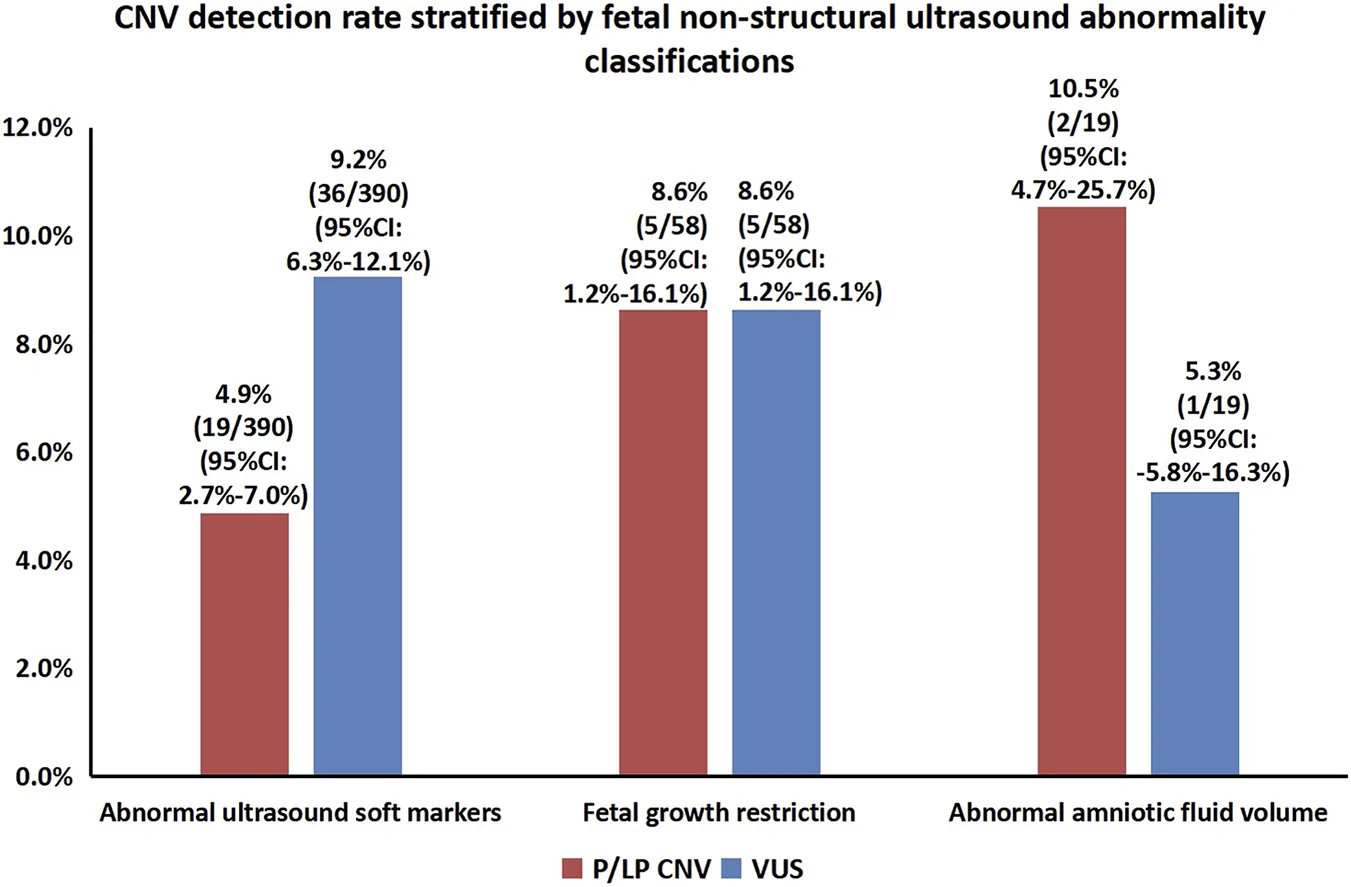
CNV detection rate stratified by fetal non-structural ultrasound abnormality classifications. Note: CNV, copy number variant; P/LP CNV, Pathogenic/Likely pathogenic CNV; VUS, variants of uncertain significance.

### Combined P/LP and VUS detection rates of different isolated ultrasound soft markers

The combined detection rate of P/LP and VUS differed among different types of isolated abnormal ultrasound soft markers, although the difference was not statistically significant (*p* = 0.153, 95% CI: 0.146–0.160) (Figure 4). For tricuspid regurgitation, the combined P/LP and VUS detection rate was 66.7% (2/3 cases); note that this estimate derives from an extremely small subgroup and bears substantial statistical uncertainty, and should not be interpreted as evidence of elevated genuine clinical genetic risk. For persistent right umbilical vein, the combined P/LP and VUS detection rate was 50.0% (1/2 cases), and for persistent left superior vena cava it was 50.0% (1/2 cases). Both estimates are based on very limited case numbers and are subject to considerable sampling fluctuation (the blue bars in Figure 4). The detection rates of other soft markers were as follows: pyelic separation 25.0% (4/16), echogenic bowel 23.1% (3/13), short long bones 21.7% (5/23), bilateral subependymal cysts 20.0% (1/5), increased NT thickness 14.8% (19/128), ventriculomegaly 11.6% (8/69), choroid plexus cyst 10.8% (4/37), and nasal bone hypoplasia 9.4% (5/53). Notably, aberrant subclavian artery and single umbilical artery showed a 0.0% CNV detection rate in this study.

**FIGURE 4:**
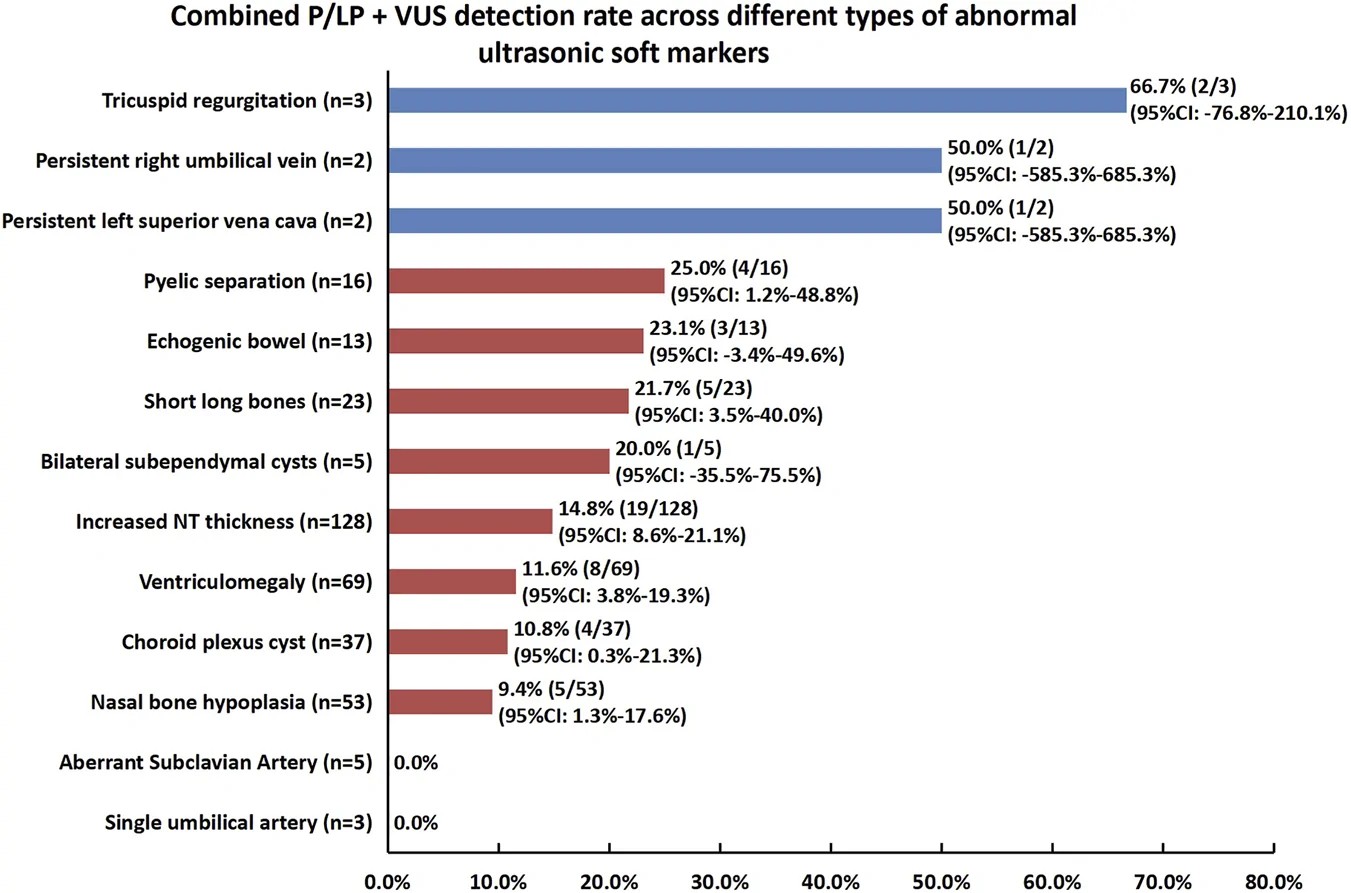
Combined P/LP and VUS detection rates across different types of abnormal ultrasound soft markers. Note: NT, nuchal translucency. Blue bar chart: These subgroup-level proportions are based on variable sample sizes; estimates from n < 5 subgroups exhibit high statistical uncertainty and are for exploratory description only, not for clinical risk ranking. Red bar chart: The types of ultrasound soft indicators abnormalities in fetuses with P/LP CNVs or VUS and ≥5 cases with these ultrasound soft markers.

### Comparison of CNV detection rates among different groups

#### Subgroup analysis of single ultrasound soft marker abnormalities

The baseline characteristics of pregnant women with and without a history of adverse pregnancy were compared (Table 3). The mean maternal age was 28.6 ± 3.6 years in the group without adverse pregnancy history and 29.3 ± 3.6 years in the group with adverse pregnancy history, showing no statistically significant difference (*p* = 0.069, 95% CI: 0.059–0.076). Similarly, the distribution of gestational age at sampling did not differ significantly between the two groups (*p* = 0.316, 95% CI: 0.307–0.326), indicating that the two groups were comparable in terms of maternal age and gestational age at sampling.

**TABLE 3:** Comparison age and the gestational age at the time of sampling between pregnant women without and with adverse obstetric history.

Characteristics	Without adverse obstetric history (n = 368)	With adverse obstetric history (n = 104)	p values	95% confidence interval
Age of pregnant women (years)	28.6 ± 3.6	29.3 ± 3.6	0.069	0.059–0.076
The gestational age at the time of sampling (weeks)	​	​	​	​
≤13, n (%)	47 (12.8%)	13 (12.5%)	0.316	0.307–0.326
14–28, n (%)	248 (67.4%)	77 (74.0%)		
>28, n (%)	73 (19.8%)	14 (13.5%)		

We further stratified participants according to the presence or absence of adverse obstetric history and compared the P/LP CNV and VUS detection rates among individual single ultrasound soft-marker subgroups (Table 1). For increased NT thickness, ventriculomegaly, nasal bone hypoplasia, choroid plexus cyst, short long bones, pyelic separation, echogenic bowel, bilateral subependymal cysts, aberrant subclavian artery, single umbilical artery, tricuspid regurgitation, and persistent left-sided superior vena cava as well as persistent right umbilical vein, we reported the number and proportion of P/LP CNVs and VUS in subgroups with and without adverse obstetric history. After Bonferroni correction for multiple comparisons, none of the between-group comparisons reached statistical significance. Of note, several subgroups contained very small sample sizes (n ≤ 3); for these subgroups, point estimates were reported together with raw numerator-denominator counts, and these estimates carry substantial statistical uncertainty and serve only for exploratory description.

### Comparison of P/LP CNVs and VUS detection between pregnant women with a history of adverse pregnancy and those without

As shown in Table 4, the detection rate of P/LP CNVs was 5.2% (19/368) in pregnant women without adverse obstetric history and 7.7% (8/104) in those with adverse obstetric history, with no statistical difference (*p* = 0.340, 95% CI: 0.336–0.355). The VUS detection rate was 9.5% (35/368) and 7.7% (8/104) in the two groups, respectively, and the difference was also not statistically significant (*p* = 0.701, 95% CI: 0.694–0.712).

**TABLE 4:** Comparison of the detection rates of fetal P/LP CNVs and VUS between pregnant women without and with adverse obstetric history, respectively.

Groups	Without adverse obstetric history (n = 368)	With adverse obstetric history (n = 104)	*p* values	95% confidence interval
P/LP CNVs	19 (5.2%)	8 (7.7%)	0.340	0.336–0.355
VUS	35 (9.5%)	8 (7.7%)	0.701	0.694–0.712

CNV, copy number variant; P/LP CNV, Pathogenic/Likely pathogenic CNV; VUS, variants of uncertain significance.

## Discussion

This retrospective study systematically analyzed the CNV screening results of 472 fetuses with non-structural ultrasound abnormalities in non-advanced maternal age pregnant women, and further explored the distribution characteristics of various non-structural ultrasonic abnormalities, the differences in CNV detection yield among different ultrasonic soft markers, as well as the correlation between maternal adverse pregnancy history and fetal P/LP CNV and VUS detection rates. The relevant findings provide practical evidence and reference basis for prenatal genetic counseling, hierarchical risk assessment and individualized clinical management in non-advanced maternal age pregnant women.

With the widespread application of high-resolution prenatal ultrasound in clinical practice, fetal non-structural ultrasound abnormalities, also defined as ultrasonic soft markers, have been frequently detected during second-trimester systematic screening (Wu et al., 2023). Different from definite fetal structural malformations, non-structural abnormalities do not form obvious anatomical malformations, but are closely correlated with chromosomal aneuploidies, microdeletion/microduplication syndromes and sporadic genetic variations (Liubing et al., 2024; Peng et al., 2024). Clinically, pregnant women aged under 35 years are traditionally classified as low-risk groups for fetal chromosomal abnormalities, and most of them rely only on serological Down’s screening for prenatal risk assessment (Li et al., 2023; Elmerdahl Frederiksen et al., 2024). However, accumulating evidence has indicated that fetal genetic abnormalities such as CNVs are not completely limited to advanced maternal age population, and a considerable proportion of pathogenic CNVs can still occur in fetuses of non-advanced age pregnant women (Li et al., 2023; Liu et al., 2024). In the present study, all enrolled participants were non-advanced maternal age pregnant women, among whom the overall detection rates of P/LP CNVs and VUS reached 5.7% and 9.1%, respectively. This result further confirms that non-advanced age cannot completely avoid the risk of fetal pathogenic CNVs (Li et al., 2023). For non-advanced age pregnant women with fetal non-structural ultrasound abnormalities, simple traditional serological screening is insufficient to exclude genetic risks, and active recommendation of chromosomal microarray-based CNV detection is clinically necessary to avoid missed diagnosis of hereditary diseases and improve the detection rate of fetal genetic abnormalities.

In this cohort, abnormal ultrasound soft markers accounted for 82.6% of all non-structural ultrasound abnormalities, occupying the absolute dominant proportion, followed by fetal growth restriction (12.3%), abnormal amniotic fluid volume (4.0%), and cases with more than two combined non-structural abnormalities (1.1%). This distribution feature is consistent with domestic and international prenatal diagnosis data, suggesting that isolated ultrasonic soft markers are the most common indication for prenatal genetic consultation in daily obstetric practice (Chen et al., 2024; Xia et al., 2020). Overall, the detection rates of CNVs varied across different categories of fetal non-structural ultrasound abnormalities, with different patterns observed between P/LP CNVs and VUS. The highest detection rate of P/LP CNVs was observed in fetuses with abnormal amniotic fluid volume, while the highest detection rate of VUS was seen in fetuses with abnormal ultrasound soft markers. Abnormal amniotic fluid volume is often closely related to placental dysfunction, fetal urinary system development abnormalities and potential genetic disorders (Jha et al., 2023; Magann et al., 2021). Fetal growth restriction is also a common phenotypic manifestation of many chromosomal microdeletion and microduplication syndromes (Chen et al., 2025). Therefore, these two types of non-structural abnormalities should be regarded as medium-to-high risk indicators in prenatal genetic counseling, and CNV detection should be prioritized. For isolated ultrasonic soft markers with relatively moderate positive rate, hierarchical counseling and personalized detection suggestions can be adopted according to the specific type of soft marker.

Further analysis of different single ultrasonic soft markers revealed that the combined detection rate of P/LP and VUS differed among different types of isolated abnormal ultrasound soft markers, although the difference was not statistically significant. Renal pyelic separation presented the highest positive rate, followed by echogenic bowel and short long bones. Given the limited sample size of other markers, we excluded them from comparative analysis. These findings suggest the above three soft markers are closely linked to increased risk of P/LP CNVs and VUS. By contrast, the CNV detection rates of choroid plexus cyst, nasal bone hypoplasia, ventriculomegaly and other common soft markers were relatively low, and isolated single occurrence usually had benign prognosis. Notably, aberrant subclavian artery and single umbilical artery showed zero CNV positive cases in this study, implying that isolated single umbilical artery and aberrant subclavian artery have low genetic correlation and can be mainly managed by regular ultrasound follow-up without excessive genetic intervention (Hu et al., 2021; Liang Y. et al., 2026). The significant differences in CNV yield among various soft markers suggest that it is inappropriate to equate all non-structural ultrasound abnormalities with identical genetic risk; clinical counselors should classify the risk level according to the specific type of soft marker, so as to avoid excessive medical intervention for low-risk markers and insufficient attention to high-risk markers.

Adverse obstetric history is an important high-risk factor for recurrent spontaneous abortion, stillbirth, fetal malformation and adverse neonatal outcomes, and it is often accompanied by potential parental chromosomal polymorphism, recessive genetic disease carriage and immune dysfunction (Yang et al., 2025). Exploring the influence of adverse obstetric history on fetal CNV detection in non-advanced age pregnant women is of great guiding value for clinical high-risk population screening. In the overall comparison of this study, the detection rates of P/LP CNVs and VUS showed no significant difference between pregnant women with and without adverse obstetric history (*p* > 0.05). It suggests that for non-advanced age pregnant women with fetal non-structural ultrasound abnormalities, maternal adverse obstetric history cannot significantly increase the overall positive rate of fetal CNVs. Although a higher observational proportion of CNV variants was observed among fetuses with echogenic bowel and concurrent adverse obstetric history, this finding was derived from a very limited sample (n = 13). Following Bonferroni correction for multiple subgroup comparisons, no statistically significant inter-group difference was detected. This observation remains hypothesis generating only and cannot support firm clinical recommendations. Definitive conclusions regarding genetic risk for fetuses presenting echogenic bowel combined with adverse obstetric history await future larger-scale independent cohort investigations. Echogenic bowel is related to fetal intestinal development disorders, intrauterine infection and genetic metabolic diseases (Vena et al., 2023). Pregnant women with adverse obstetric history may have underlying genetic susceptibility or intrauterine adverse environmental factors, which may further increase the genetic abnormal risk of fetuses (Xue et al., 2024; Lee et al., 2021). However, the research conducted by Ronin et al. revealed that the ultrasound grading of the fetal echogenic bowel was not a predictor of the neonatal outcome (Ronin et al., 2017). Therefore, the relationship between this ultrasound soft marker and the fetal prognosis still requires further research to be clarified.

VUS accounted for 9.1% of the total cases in this study, which is consistent with the VUS proportion reported in most prenatal CNV detection studies (Song et al., 2019; Mastromoro et al., 2022), and is slightly higher than that in some other studies (Mardy et al., 2021; Li et al., 2024; Huang et al., 2021). Due to the limitations of current genomic database annotation, incomplete phenotypic-gene correlation data and racial difference in genetic variation distribution, VUS cannot be accurately classified as pathogenic or benign at this stage (Cornthwaite et al., 2022). The emergence of VUS often brings huge psychological pressure and decision-making dilemma to pregnant women and their families. In clinical practice, clinicians should avoid exaggerated interpretation and excessive clinical intervention of VUS results, fully inform the parents of the uncertainty of VUS, recommend regular prenatal ultrasound follow-up, and conduct postnatal physical examination and developmental assessment. With the continuous update of genomic databases and the accumulation of clinical follow-up data, the clinical significance of most VUS will be gradually clarified in the future.

This study has several limitations. First, this was a single-center retrospective observational study recruiting consecutive eligible cases; therefore, formal prospective sample-size calculation and power analysis were not performed before data collection. Specially, the observed 5.7% P/LP CNV rate is restricted to non-advanced maternal age pregnant women referred for invasive prenatal testing because of fetal non-structural ultrasound abnormalities and cannot be extrapolated to all pregnant women aged <35 years or all patients presenting the same ultrasound features. Some rare ultrasound soft-marker subgroups contained relatively small numbers of participants, which might produce statistical bias and restrict the generalizability of our findings. Second, considering the low absolute number of P/LP-CNV positive events in several stratified subgroups, the events-per-variable (EPV) requirement for stable multivariate logistic regression could not be satisfied; implementing multivariate logistic regression under such conditions would carry a high risk of model overfitting and yield unreliable coefficient estimates. And multiple statistical tests were conducted for different ultrasound soft-marker subgroups in this study. Formal correction for multiple comparisons was not performed, which may increase the risk of type I error. Accordingly, all subgroup-level statistical results should be interpreted only as exploratory, hypothesis-generating findings, and cannot serve as conclusive clinical evidence. So, these potential associations need to be validated in larger independent cohorts. Third, this study lacked long-term postnatal follow-up of CNV-positive and VUS fetuses, and could not further verify the consistency between genetic variation genotype and postnatal phenotypic performance. Fourth, we did not conduct parental genetic verification for CNV-positive cases, which cannot distinguish *de novo* variation from inherited variation. In addition, adverse obstetric history was treated as a composite variable combining recurrent miscarriage, stillbirth, neonatal death and malformed birth. Owing to limited case numbers for each individual subtype, stratified analysis by specific obstetric-history category could not be performed. Pooling these etiologically heterogeneous events may reduce statistical power and mask potential subtype-specific associations with fetal CNV risk. Another limitation relates to the interpretation of subgroup-specific detection rates for fetal ultrasound soft markers. The rates presented in Figure 4 represent the combined P/LP + VUS detection rate, which includes VUS. As defined in our Methods section, P/LP CNVs serve as the primary clinical endpoint, whereas VUS are only exploratory outcomes. This combined metric does not directly reflect true pathogenic genetic risk.

Standardized prenatal counseling and postnatal follow-up are essential for fetuses with VUS. Clinicians should avoid over-interpreting pathogenic risk and fully inform families of the uncertainty of VUS caused by incomplete genomic database evidence. Serial prenatal ultrasound monitoring is recommended, together with standardized neonatal physical and neurodevelopmental assessment after birth. Drawing on our results and existing evidence, we propose tiered prenatal genetic counseling protocols for non-advanced-age pregnant women with fetal non-structural ultrasound abnormalities. For high-risk features including abnormal amniotic fluid volume, fetal growth restriction, and high-yield soft markers (pyelic separation, echogenic bowel, short long bones), CMA is strongly recommended regardless of adverse obstetric history. For intermediate-risk isolated soft markers, CMA should be considered combined with adverse obstetric history. For low-risk markers such as isolated aberrant subclavian artery and single umbilical artery, routine ultrasound follow-up is preferred, with invasive testing optional after adequate counseling.

## Conclusion

In conclusion, non-advanced age pregnant women with fetal non-structural ultrasound abnormalities still face measurable risks of pathogenic/likely-pathogenic CNVs and VUS. Distinct CNV detection rates exist across different non-structural ultrasound abnormalities and isolated soft markers. Based on data from the overall cohort, maternal adverse obstetric history does not alter the global fetal CNV detection rate. Nevertheless, these findings require further validation in larger cohorts. Clinically, a stratified risk-assessment system for fetal non-structural ultrasound abnormalities should be implemented. Combined consideration of maternal age, adverse obstetric history and specific ultrasound markers supports individualized prenatal genetic strategies.

## Data Availability

The data presented in the study are available from the corresponding author upon reasonable request and with approval from the institutional ethics committee.
