# Supplementary material for: Study on copy number variant detection results of 472 fetuses with non-structural ultrasound abnormalities in non-advanced age pregnant women and the correlation with adverse obstetric history
Source: Front Genet. 2026 Oct 30;17:1895948. doi: 10.3389/fgene.2026.1895948 (PMC13760234; doi:10.3389/fgene.2026.1895948)
Supplement: Supplementary file 1 [file Table1.docx]

Supplementary Table 1 The clinical data of 27 fetuses with P/LP CNVs were detected by CMA analysis.

| Number | Age of pregnant woman (years) | The gestational age at the time of sampling (weeks) | Types of ultrasound abnormalities | Types of fetuses samples | CMA results | Clinical significance of CNVs |
| --- | --- | --- | --- | --- | --- | --- |
| 1 | 23 | 17^+3^ | Tricuspid regurgitation | Amniotic fluid | arr[GRCh37]7q11.23(72701099-74154209)×1 (1.45Mb) | P |
| 2 | 33 | 13^+2^ | Increased NT thickeness, single umbilical artery | Villu | arr[GRCh37]10q26.11q26.3(121223476-135426386)×1 (14.20Mb) | P |
| 3 | 23 | 28^+6^ | Ventriculomegaly | Amniotic fluid | arr[GRCh37]Xp22.31(6449837-8143509)×1 (1.69Mb) | P |
| 4 | 26 | 26^+4^ | Fetal growth restriction | Amniotic fluid | arr[GRCh37]22q11.21(18631365-21800471)×1 (3.17Mb) | P |
| 5 | 30 | 17^+2^ | Tricuspid regurgitation | Amniotic fluid | arr[GRCh37]Xp21.1(31750507-31889320)×1 (139Kb) | P |
| 6 | 29 | 19^+4^ | Choroid plexus cyst | Amniotic fluid | arr[GRCh37]8p23.2p22(2347605-17861146)×3 (15.51Mb) | P |
| 7 | 26 | 21^+5^ | Pyelic separation | Amniotic fluid | arr[GRCh37]Xp21.1(31713418-31825390)×0 (112Kb) | P |
| 8 | 27 | 13 | Increased NT thickeness | Villu | arr[GRCh37]3q13.31q22.1(116764010-129606827)×2 hmz (12.84Mb) | LP |
| 9 | 26 | 12^+6^ | Increased NT thickeness | Villu | arr[GRCh37]16p11.2(29428532_30190029)x1 (761Kb) | P |
| 10 | 25 | 18^+2^ | Ventriculomegaly | Amniotic fluid | arr[GRCh37]Xp22.31(6455152_8135568)x1 (1.68Mb) | P |
| 11 | 29 | 12^+2^ | Increased NT thickeness | Villu | arr[GRCh37]16p13.11(15338153_16327887)x3 (990Kb) | LP |
| 12 | 28 | 32^+2^ | Fetal growth restriction | Amniotic fluid | arr[GRCh37]15q11.2(22770422_23288350)x1 (518Kb) | P |
| 13 | 29 | 18^+1^ | Increased NT thickeness, Fetal growth restriction | Amniotic fluid | arr[GRCh37]Xp22.31(6455152_8135568)x1 (1.68Mb) | P |
| 14 | 30 | 27 | Ventriculomegaly | Amniotic fluid | arr[GRCh37]7q11.23(72682113_74030629)x3 (1.35Mb) | P |
| 15 | 27 | 19^+6^ | Nasal bone hypoplasia | Amniotic fluid | arr[GRCh37]15q11.2(22770422_23082237)x1 (312Kb) | P |
| 16 | 27 | 25^+3^ | Polyhydramnios | Amniotic fluid | arr[GRCh37]15q11.2(22770422_23214655)x1 (444Kb) | P |
| 17 | 31 | 35^+6^ | Fetal growth restriction | Amniotic fluid | arr[GRCh37]15q11.2q13.1(23290788_28540345)x1 (5.25Mb) | P |
| 18 | 25 | 18^+4^ | Increased NT thickeness | Amniotic fluid | arr[GRCh37]15q11.2(22770422_23288350)x1 (518Kb) | P |
| 19 | 21 | 24^+6^ | Echogenic bowel | Amniotic fluid | arr[GRCh37]15q11.2(22770422_23082237)x1 (312Kb) | P |
| 20 | 28 | 31^+3^ | Short long bones | Amniotic fluid | arr[GRCh37]2q13(111371702_113111856)x1 (1.74Mb) | LP |
| 21 | 26 | 29 | Ventriculomegaly | Amniotic fluid | arr[GRCh37]16p11.2(28708187_29090030)x1 (382Kb) | P |
| 22 | 28 | 12^+6^ | Increased NT thickeness | Villu | arr[GRCh37]15q11.2(22770422_23282798)x1 (512Kb) | P |
| 23 | 33 | 24^+2^ | Nasal bone hypoplasia | Amniotic fluid | arr[GRCh37]15q21.3q26.3(56282641_102429040)x2~3mos (46.15Mb) | P |
| 24 | 28 | 17^+4^ | Increased NT thickeness | Amniotic fluid | arr[GRCh37]15q11.2(22770422_23082237)x1 (312Kb) | P |
| 25 | 34 | 32^+4^ | Fetal growth restriction | Amniotic fluid | arr[GRCh37]15q11.2(22770422_23282798)x1 (512Kb) | P |
| 26 | 29 | 32^+1^ | Polyhydramnios | Amniotic fluid | arr[GRCh37] 22q11.21(18631365_21800471)x1 (3.17Mb) | P |
| 27 | 28 | 32^+2^ | Fetal growth restriction | Amniotic fluid | arr[GRCh37]Xp22.31(6578896_7918931)x0 (1.34Mb) | P |

P/LP CNV, Pathogenic/Likely pathogenic CNV
