# Supplementary material for: Study on copy number variant detection results of 472 fetuses with non-structural ultrasound abnormalities in non-advanced age pregnant women and the correlation with adverse obstetric history
Source: Front Genet. 2026 Oct 30;17:1895948. doi: 10.3389/fgene.2026.1895948 (PMC13760234; doi:10.3389/fgene.2026.1895948)
Supplement: Supplementary file 2 [file Table2.docx]

Supplementary Table 2 The clinical data of 43 fetuses with VUS were detected by CMA analysis.

| Number | Age of pregnant woman (years) | The gestational age at the time of sampling (weeks) | Types of ultrasound abnormalities | Types of fetuses samples | CMA results | Clinical significance of CNVs |
| --- | --- | --- | --- | --- | --- | --- |
| 1 | 32 | 31 | Fetal growth restriction | Amniotic fluid | arr[GRCh37]3q11.2q12.1(95527472-98345123)×3 (2.82Mb) | VUS |
| 2 | 26 | 31^+5^ | Ventriculomegaly | Amniotic fluid | arr[GRCh37]16p12.2(21405328-21816543)×1 (411Kb) | VUS |
| 3 | 25 | 12^+5^ | Increased NT thickeness | Villu | arr[GRCh37]22q11.21q11.23(21804597-23654007)×3 (1.85Mb) | VUS |
| 4 | 31 | 16^+3^ | Increased NT thickeness | Amniotic fluid | arr[GRCh37]4q12q13.1(58193592-62730657)×3 (4.54Mb) | VUS |
| 5 | 33 | 18 | Nasal bone hypoplasia | Amniotic fluid | arr[GRCh37]15q15.3(43868571_44007537)x1 (139Kb) | VUS |
| 6 | 33 | 12 | Increased NT thickeness | Villu | arr[GRCh37]5q14.2(81492684_82000597)x3 (508Kb) | VUS |
| 7 | 30 | 28 | Ventriculomegaly | Amniotic fluid | arr[GRCh37]2q13(110498142_110980295)x3 (482Kb) | VUS |
| 8 | 27 | 30^+4^ | Fetal growth restriction | Amniotic fluid | arr[GRCh37]4q35.2(187929332_188943890)x3 (1.02Mb) | VUS |
| 9 | 29 | 26^+4^ | Short long bones | Amniotic fluid | arr[GRCh37] 6q27(169848646_170270972)x3 (422Kb) | VUS |
| 10 | 26 | 12 | Increased NT thickeness | Villu | arr[GRCh37] 5q35.3(178730385_178936592)x3 (206Kb) | VUS |
| 11 | 26 | 30 | Fetal growth restriction | Amniotic fluid | arr[GRCh37] 1p36.33(1413423_2059598)x3 (646Kb) arr[GRCh37] 2q21.1(130926994_131194508)x3 (268Kb) | VUS |
| 12 | 24 | 24 | Bilateral subependymal cysts | Amniotic fluid | arr[GRCh37] Xq28(148565633_148884123)x3 (318Kb) | VUS |
| 13 | 27 | 19 | Pyelic separation | Amniotic fluid | arr[GRCh37]22q11.22(22311349_22579064)x1 (268Kb) | VUS |
| 14 | 32 | 20^+5^ | Increased NT thickeness | Amniotic fluid | arr[GRCh37]15q11.2(22770422_23625785)x3 (855Kb) arr[GRCh37]Xq28(151872967_152087536)x3 (215Kb） | VUS |
| 15 | 29 | 12^+5^ | Increased NT thickeness | Villu | arr[GRCh37]15q15.3(43868571_44007537)x1 (139Kb) | VUS |
| 16 | 20 | 28^+2^ | Polyhydramnios | Amniotic fluid | arr[GRCh37]21q22.13(38126288_38637729)x3 (511Kb) arr[GRCh37]4q25q32.3(113768426_167713699)x2 hmz (53.95Mb) arr[GRCh37]4q11q22.1(52696792_89739479)x2 hmz (37.04Mb) arr[GRCh37]4p15.1p11(31236026_49063479)x2 hmz (17.83Mb) | VUS |
| 17 | 27 | 12^+6^ | Increased NT thickeness | Villu | arr[GRCh37]2q24.1q31.1(159577115_177258204)x2 hmz (17.68Mb) arr[GRCh37]16p13.3p12.3(94808_17409998)x2 hmz (17.32Mb) | VUS |
| 18 | 34 | 29 | Single umbilical artery, Fetal growth restriction | Amniotic fluid | arr[GRCh37]11q24.3q25(130391533_131249323)x1 (858Kb) | VUS |
| 19 | 28 | 24^+6^ | Nasal bone hypoplasia | Amniotic fluid | arr[GRCh37]12q21.31(82919621_86264470)x3 (3.34Mb) | VUS |
| 20 | 31 | 19^+2^ | Increased NT thickeness | Amniotic fluid | arr[GRCh37]15q26.2q26.3(97754343_98997760)x3 (1.24Mb) | VUS |
| 21 | 27 | 17 | Increased NT thickeness | Amniotic fluid | arr[GRCh37]Xp22.33(1358802_3520663)x3 (2.16 Mb) | VUS |
| 22 | 30 | 18 | Echogenic bowel | Amniotic fluid | arr[GRCh37]18q22.1(63879972_66569034)x3 (2.69Mb) | VUS |
| 23 | 32 | 29^+1^ | Short long bones | Amniotic fluid | arr[GRCh37]10q26.3(134639293_135426386)x1 (787Kb) | VUS |
| 24 | 26 | 27^+4^ | Ventriculomegaly | Amniotic fluid | arr[GRCh37]Yp11.2(6135281_9171486)x0 (3.04Mb) | VUS |
| 25 | 27 | 27 | Fetal growth restriction | Amniotic fluid | arr[GRCh37]1p36.33p11.2(888659_121339317)x2 hmz (120.45 Mb) arr[GRCh37]1q21.2q44(149879545_249198164)x2 hmz (99.32 Mb) | VUS |
| 26 | 27 | 31^+1^ | Pyelic separation | Amniotic fluid | arr[GRCh37]22q11.23(23652587_25041592)x3 (1.39 Mb) | VUS |
| 27 | 27 | 12^+2^ | Increased NT thickeness | Villu | arr[GRCh37]12p11.1(33430047_34599314)x3 (1.17Mb) | VUS |
| 28 | 28 | 16^+5^ | Increased NT thickeness | Amniotic fluid | arr[GRCh37]1q44(248003188_248771545)x1 (768Kb) | VUS |
| 29 | 32 | 25^+2^ | Persistent left superior vena cava | Amniotic fluid | arr[GRCh37]1q21.1(145406788_145888926)x3 (482Kb) | VUS |
| 30 | 30 | 30^+4^ | Fetal growth restriction | Amniotic fluid | arr[GRCh37]4q35.2(187933402_188943890)x3 (1.01Mb) arr[GRCh37]5q23.1(116670621_118148274)x3 (1.48Mb) | VUS |
| 31 | 32 | 13^+3^ | Increased NT thickeness | Villu | arr[GRCh37]1p36.11(24360737_24867196)x1 (506Kb) arr[GRCh37]4q12q13.1(58221562_62730657)x3 (4.51Mb) | VUS |
| 32 | 33 | 17^+1^ | Choroid plexus cyst | Amniotic fluid | arr[GRCh37]19q12(29568991_30605808)x1 (1.04 Mb) | VUS |
| 33 | 28 | 25 | Short long bones | Amniotic fluid | arr[GRCh37]4q23(99562179_100431609)x1 (869 Kb) | VUS |
| 34 | 22 | 27^+6^ | Short long bones | Amniotic fluid | arr[GRCh37]16p13.3p11.2(94808_31935367)x2 hmz (31.84Mb) | VUS |
| 35 | 30 | 19^+6^ | Choroid plexus cyst | Amniotic fluid | arr[GRCh37]12p11.21(31742092_32280459)x1 (538Kb) | VUS |
| 36 | 26 | 23^+1^ | Persistent right umbilical vein | Amniotic fluid | arr[GRCh37]18q12.1(26834461_28442180)x3 (1.61Mb) | VUS |
| 37 | 33 | 12 | Increased NT thickeness | Villu | arr[GRCh37]21q22.13q22.2(38093453_40120580)x3 (2.03Mb) arr[GRCh37]Xp21.1(33850092_34789493)x0 (939Kb) | VUS |
| 38 | 28 | 18^+6^ | Increased NT thickeness, Choroid plexus cyst | Amniotic fluid | arr[GRCh37]13q31.1(80376841_84148560)x1 (3.77Mb) | VUS |
| 39 | 31 | 18 | Echogenic bowel | Amniotic fluid | arr[GRCh37]4q35.2(187933402_188943890)x3 (1.01Mb) | VUS |
| 40 | 24 | 25^+6^ | Ventriculomegaly | Amniotic fluid | arr[GRCh37]8p23.3(1125565_2193914)x3 (1.07Mb) | VUS |
| 41 | 29 | 17^+1^ | Choroid plexus cyst | Amniotic fluid | arr[GRCh37]19q12q13.33(30841000_51279882)x2 hmz (20.44Mb) | VUS |
| 42 | 29 | 22^+2^ | Nasal bone hypoplasia | Amniotic fluid | arr[GRCh37]7p21.3p15.2(8745176_26439331)x2 hmz (17.69Mb) arr[GRCh37]20q11.21q13.12(29510307_45556516)x2 hmz (16.05Mb) | VUS |
| 43 | 29 | 18^+3^ | Pyelic separation | Amniotic fluid | arr[GRCh37]21q21.1(22372647_22982857)x1 (610Kb) | VUS |

VUS, variants of uncertain significance.
